# Wiring the Brain by Clustered Protocadherin Neural Codes

**DOI:** 10.1007/s12264-020-00578-4

**Published:** 2020-09-17

**Authors:** Qiang Wu, Zhilian Jia

**Affiliations:** Center for Comparative Biomedicine, Ministry of Education Key Lab of Systems Biomedicine, State Key Laboratory of Oncogenes and Related Genes, Joint International Research Laboratory of Metabolic and Developmental Sciences, Institute of Systems Biomedicine, Xinhua Hospital, School of Life Sciences and Biotechnology, Shanghai Jiao Tong University, Shanghai, 200240 China

**Keywords:** Clustered protocadherins, Genome architecture, Neuronal identity, Adhesion specificity, Self-avoidance, Cell recognition

## Abstract

There are more than a thousand trillion specific synaptic connections in the human brain and over a million new specific connections are formed every second during the early years of life. The assembly of these staggeringly complex neuronal circuits requires specific cell-surface molecular tags to endow each neuron with a unique identity code to discriminate self from non-self. The clustered protocadherin (*Pcdh*) genes, which encode a tremendous diversity of cell-surface assemblies, are candidates for neuronal identity tags. We describe the adaptive evolution, genomic structure, and regulation of expression of the clustered *Pcdh*s. We specifically focus on the emerging 3-D architectural and biophysical mechanisms that generate an enormous number of diverse cell-surface Pcdhs as neural codes in the brain.

## Introduction

The human brain contains a staggering 86 billion neurons, each with numerous branches of dendrites covering receptive fields and of axons innervating diverse regions with minimal overlap. The correct patterning of dendritic and axonal arbors is central for establishing and maintaining enormously complex networks with specific neuronal connectivity in the brain. These vast networks of synaptic connections between axons and dendrites form specific neuronal circuits to fulfill complicated cognitive functions and to determine personality traits and behavior. Aberrant assemblies of neuronal circuits underlie neuropsychiatric diseases. Neuronal circuit assemblies require each neuron to have an identity code for self-recognition and non-self discrimination. How these fascinating and diverse neuronal networks are generated is of the utmost importance. In addition, how the limited size of the human genome encodes the enormous number of neuronal cell-surface identity codes is intriguing.

Over the past few decades, great progress has been made to uncover large families of adhesion proteins that are candidates for cell-surface identity codes for neuronal circuit assembly, such as neurexins [1], olfactory receptors [2], cadherins and families of other adhesion molecules [3–6]. For example, in *Drosophila melanogaster*, 38,016 isoforms of *Dscam1* (Down syndrome cell adhesion molecule 1)—generated by alternative splicing—endow each neuron with a unique identity code to discriminate self from non-self [7–10]. In vertebrates, this is achieved through the stochastic and combinatorial expression of ~60 clustered protocadherin (*Pcdh*) genes [11–13].

Cadherins are a superfamily of Ca^2+^-dependent cell-adhesion proteins that are required for specific cell-cell recognition in metazoans. Members of the cadherin superfamily include classical cadherins (type I and type II), clustered Pcdhs (α, β, γ), and non-clustered Pcdhs [6]. Compared with classical cadherins with five ectodomains (ECs), Pcdhs have six or more ECs with characteristic genome organization, in which multiple ECs are encoded by single unusually large exons [14, 15], and have diverse functions such as neuronal migration and axonal development [15, 16]. Clustered *Pcdh* genes are arranged in closely-linked clusters in one chromosomal region, while non-clustered *Pcdh* genes are scattered on different chromosomes [17]. As the largest subfamily of the cadherin superfamily, clustered *Pcdh* genes are prominently expressed in the brain, and each encodes a cadherin-like protein with six characteristic EC repeats. Their variable and constant genomic architectures are remarkably similar to those of the immunoglobulin (*Ig*), T cell receptor *(Tcr)*, and UDP glucuronosyltransferase (*Ugt*) gene clusters, which generate tremendous diversity for the humoral immunity, cellular immunity, and chemical defense systems, respectively [11, 18].

In this review, we describe 3-D architectural and biophysical mechanisms for Pcdh neural codes in the brain. We first describe the 1-D genomic organization of the three *Pcdh* gene clusters and the 3-D architectural mechanisms that generate their combinatorial repertoires for single neurons. We then discuss *cis*- and *trans*-interactions between the extracellular domains of cell-surface Pcdh proteins to ensure neurons for self-recognition as well as self and non-self discrimination. These interactions transduce extracellular contact-dependent signals into the cytoplasm to induce actin dynamics and cytoskeletal remodeling through the common intracellular constant domains. It is this cytoskeletal remodeling that leads to the many functions of Pcdh such as neuronal migration, neurite morphogenesis, dendritic self-avoidance, axonal projection, spine elaboration, synaptogenesis, and neuronal connectivity. We refer interested readers to other excellent reviews discussing various aspects of the clustered *Pcdh* genes [5, 6, 19–25].

## If It Looks Like a Code and Organizes Like a Code, It is a Code

Genetic studies have a long history of describing the phenomena of heredity. While individual genes determine certain phenotypes, the genome with the entire gene assembly holds the characteristics of a species and every creature has a genome that is passed on to the next generation. The genome encodes the brain, but the environment shapes and sharpens the brain: so-called neural epigenetics. The complexity of the brain determines the mind and consciousness. Both the brain and genome code and store information that is vital for the life of creatures. While the genome and genetic codes have been decoded [26, 27], the nature of the neural codes that wire the brain is still under intense investigation.

### Setting the Stage for Neural Identity Codes

In the early 1940s, the Chemoaffinity Hypothesis posited that neurons express on their plasma membranes individual identification tags that specify synaptic connections [28]. Intensive efforts have since been devoted to uncovering the proposed neural codes but the exact nature of the neuronal chemoaffinity tags remains elusive [29, 30]. Among the four cell-adhesion families of cadherins, selectins, integrins, and Ig-containing proteins, cadherins are the only family that functions in direct Ca^2+^-dependent plasma membrane-to-membrane homotypic interactions, and are thus strong candidates for the chemoaffinity tags of neural codes in the brain [3, 5, 6, 31, 32]. However, only about a dozen classical cadherin genes and a few *Pcdh* genes were cloned in the nineties [33, 34]. Using the yeast two-hybrid system, 2 full-length and 6 partial cadherin-related receptor genes were cloned from mouse brain tissues and found to be expressed at synaptic junctions in neuronal subpopulations [35]. However, where exactly these proteins are located remains to be determined.

It turned out that these genes are members of the *Pcdhα* cluster which happens to be located upstream of the two other large gene clusters of *Pcdhβ* and *Pcdhγ* [11]. In total, there are 15 *Pcdhα*, 16 *Pcdhβ*, and 22 *Pcdhγ* genes that are highly similar and organized in tandem arrays in a single locus of the human genome. These large numbers and the striking organization immediately suggest that the clustered *Pcdh* genes are the long-sought neuronal address codes for the brain [4, 36–38]. These numbers are orders of magnitude less than that of neurons in the brain; however, mathematic analyses suggest that they are enough to encode the synaptic address codes required for geometrically constrained local brain regions or nuclei [39].

### Genomic Organization of Clustered *Pcdh* Genes

**Fig. 1:**
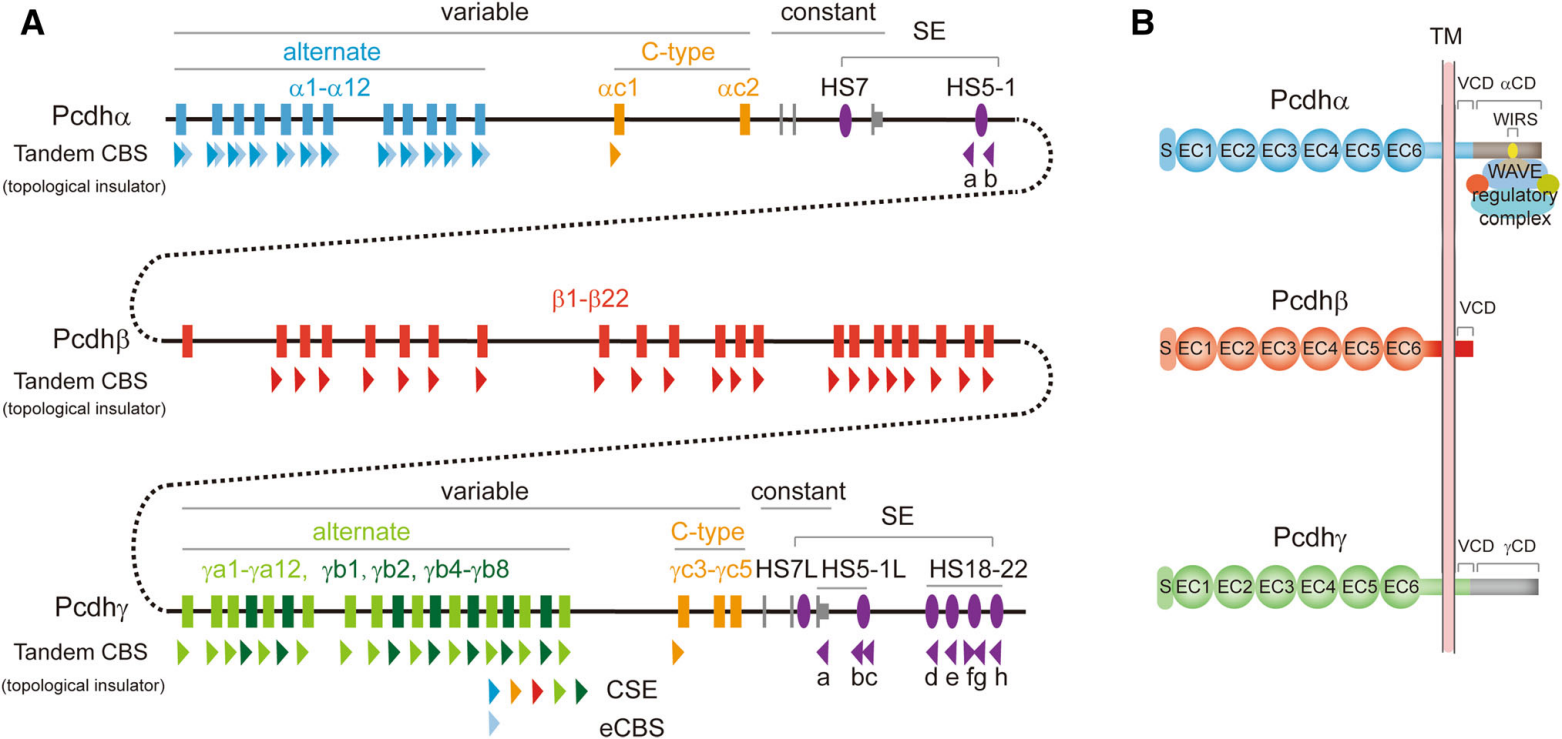
Genomic organization and domain structure of clustered protocadherins. **A** Mouse clustered protocadherin genes have 58 isoforms arranged into three closely-linked clusters: *Pcdh α*, *β*, and *γ*. The *Pcdh α* and *γ* gene clusters contain more than a dozen of unusually large, highly similar, and repetitive variable exons, each of which is associated with a promoter and can be spliced to a common set of three downstream small constant exons within the respective cluster. These variable exons can be separated into alternate and C-type groups, based on the encoded protein sequence similarity. The *Pcdhβ* gene cluster lacks constant exons and only contains 22 variable exons each of which encodes a full-length protein. HS7 and HS5-1 constitute a super-enhancer (SE) for the *Pcdhα* cluster. HS7L, HS5-1L, and HS18-22 constitute a super-enhancer for the *Pcdh β* and *γ* clusters. The locations and relative orientations of tandem CTCF sites (CBS, CTCF binding site), which function as topological insulators, are marked as arrowheads under the respective promoters and enhancers. Note that each *Pcdhα* alternate promoter is flanked by two CBS elements (CSE and eCBS). HS, DNaseI hypersensitive site. **B** The domain organization of the encoded protein structure of clustered Pcdhs. Each large variable exon encodes an extracellular domain with a signal peptide, followed by 6 ectodomain (EC) repeats, a transmembrane (TM) domain, and a juxtamembrane variable cytoplasmic domain (VCD). The three small constant exons encode a common membrane-distal intracellular constant domain (CD) shared by all isoforms of the *Pcdh α* or *γ* cluster. There is a WAVE interacting receptor sequence (WIRS) motif located near the C-terminal end of the *Pcdhα* CD that recruits the WAVE-regulatory complex and links to actin cytoskeletal dynamics.

The mammalian clustered Pcdh proteins are encoded by three closely-linked gene clusters (*Pcdhα*, *Pcdhβ*, and *Pcdhγ*) which span nearly 1 million base pairs [11]. The genomic arrangements of the *Pcdhα* and *Pcdhγ* clusters are similar, both with tandem arrays of large variable exons followed by respective single sets of three small constant exons (Fig. 1A) [11, 14, 40]. Within the *Pcdhα* and *Pcdhγ* clusters, each variable exon carries its own promoter and can be spliced to the single set of downstream constant exons of its respective cluster. Through stochastic promoter activation and *cis*-alternative splicing, clustered *Pcdh*s can generate dozens of different isoforms [41, 42].

The variable exons of *Pcdhα* and *Pcdhγ* can be further divided into alternate and C-type gene groups based on their genomic location and sequence similarity (Fig. 1A). The mouse *Pcdhα* cluster contains 12 alternate genes (*α1–α12*) and two C-type genes (*αc1* and *αc2*). The mouse *Pcdhγ* cluster contains 19 alternate genes (12 A-types: *γa1–γa12*; 7 B-types: *γb1*, *γb2*, *γb4–γb8*) and three C-type genes (*γc3–γc5*). Different from *Pcdhα* and *Pcdhγ*, the mouse *Pcdhβ* cluster, however, contains 22 genes (*β1–β22*) and no C-type gene (Fig. 1A). In total, there are five C-type variable exons that are more similar to each other than to members of the alternate gene group [11, 40]. However, *Pcdhβ* contains only large variable exons and lacks constant exons (Fig. 1A). Therefore, each member of the *Pcdhβ* cluster is a single-exon gene [11, 40]. Together, these three clusters encode 58 Pcdh isoforms (14α, 22β, and 22γ) in mice and 53 Pcdh isoforms (15α, 16β, and 22γ) in humans (Fig. 1A).

In the *Pcdhα* cluster, the promoter of each alternate gene is flanked by two CTCF-binding sites (CBS or CTCF sites). In the *Pcdhβ* cluster, the promoter of each gene is associated with one CBS element except *β1* which has no CBS element (Fig. 1A). In the *Pcdhγ* cluster, the promoter of each alternate gene is associated with one CBS element. Finally, among the five C-type *Pcdh* genes, only the first C-type gene of the *Pcdhα* cluster (*αc1*) and the first C-type gene of the *Pcdhγ* cluster (*γc3*) are associated with a CBS element (Fig. 1A).

Each variable exon encodes a signal peptide, followed by an extracellular domain containing 6 ECs, a transmembrane region, and a juxtamembrane variable cytoplasmic domain (VCD). The three constant exons encode a common membrane-distal intracellular constant domain (CD) shared by all members of the *Pcdhα* or *Pcdhγ* family. Since the *Pcdhβ* cluster has only a variable region with no constant region, each *Pcdhβ* variable exon is an independent gene, which encodes a Pcdh protein with an extracellular domain of 6 ECs, a transmembrane region, and a short VCD, but lacks a common CD (Fig. 1B) [11, 14].

The *Pcdha* cluster is regulated by a super-enhancer composed of two *cis*-regulatory elements, *HS7* and *HS5-1* (HS, hypersensitive site) (Fig. 1A). Similarly, a super-enhancer, composed of *HS7L* (HS7 like), *HS5-1L* (HS5-1 like), and *HS18-22*, was also identified downstream of the *Pcdhγ* cluster for both the *Pcdhβ* and *Pcdhγ* clusters (Fig. 1A) [43–48].

Fifteen DNaseI hypersensitive sites (*HS15-HS1*) were initially identified in the *Pcdhα* cluster, among which *HS7* and *HS5-1* have strong enhancer activity in a transgenic reporter assay [49]. In mice, genetic deletion of *HS5-1*, which is located 30 kb downstream of the last *Pcdhα* constant exon, results in a significant decrease in the expression levels of *Pcdhα1*–α*12* and *Pcdhαc1* in the brain, but does not affect the expression of *Pcdhαc2* [48, 50]. By contrast, deletion of *HS7*, which is located between the constant exons 2 and 3, results in a significant decrease of expression levels of all *Pcdhα* genes, including *Pcdhαc2* [50].

### Adaptive Evolution of Clustered *Pcdh* Genes

Initial studies on *Pcdh* genes showed that the encoded extracellular domain contains a “primordial” cadherin motif, similar to cadherin motifs in the *Drosophila* Fat protein [34]. It was thought that Pcdhs may be evolutionarily more ancient than the classical cadherins [34]. In addition, the *Pcdh* genes have characteristic genomic organizations in which multiple ECs are encoded by large exons, a feature that is distinct from the genomic organizations of classical cadherins [14]. Complete sequencing of the *Drosophila* genome revealed, however, that it does not contain clustered *Pcdh* genes [51]. Thus, the “proto” affix in the “protocadherin” nomenclature is a misnomer and the clustered *Pcdh* genes are thought to have adaptively evolved later and may be related to functions of more advanced nervous systems.

Similar to the human genome, the chimpanzee, mouse, and rat genomes contain the three *Pcdh* gene clusters [40, 52, 53]. Clustered *Pcdh* genes also exist in the anole lizard, frog, coelacanth, fugu, and zebrafish [52, 54–59]. The genome of the frog *Xenopus tropicalis* contains the *Pcdh α* and *γ* clusters but lacks *Pcdhβ*; however, the *Pcdhγ* cluster has been duplicated into two clusters [59]. In addition, the fugu and zebrafish genomes lack the *Pcdhβ* cluster but contain two *Pcdh α* and *γ* clusters because of the whole-genome duplication in the ray-finned lineage [52, 54, 57].

The anole and coelacanth genomes contain the *Pcdhβ* cluster [55, 58]. This suggests that the *Pcdhβ* cluster in mammals, anole, and coelacanth probably results from the duplication of variable exons of the *Pcdhγ* cluster. The duplicated variable exons subsequently lost their ability to be spliced to the constant exon 1 of the *Pcdhγ* cluster. Another possibility is that the *Pcdhβ* cluster results from duplication of the entire *Pcdhγ* cluster. The duplicated cluster then lost its constant exons through mutation or degeneration. Further research is needed to distinguish these two scenarios. Nevertheless, the topological regulation of both *Pcdh β* and *γ* clusters by a single super-enhancer composed of tandem arrays of CTCF sites (Fig. 1A) suggests that they share a common ancestor [48], consistent with their evolutionary trees [52]. Finally, molecular and structural analyses revealed that *Pcdhβ* and *Pcdhγ* share characteristics that are distinct from *Pcdhα* [12, 60].

The cartilaginous shark genome contains a single locus composed of four closely-linked *Pcdh* clusters that are para-orthologous to the three mammalian *Pcdh* gene clusters, suggesting that the ancestral jawed vertebrates contained seven *Pcdh* gene clusters [61]. During the evolution of the genomes of cartilaginous fish and bony vertebrates, this ancestral *Pcdh* locus experienced differential losses in that the mammalian lineages lost four clusters and the shark lineage lost three clusters [61]. Interestingly, clustered *Pcdh* genes are vastly expanded in the invertebrate octopus genome and enriched in neural tissues, consistent with their roles in establishing and maintaining the large and complex octopus nervous system [62, 63].

## 3-D Genome Architecture of Clustered *Pcdhs*

**Fig. 2:**
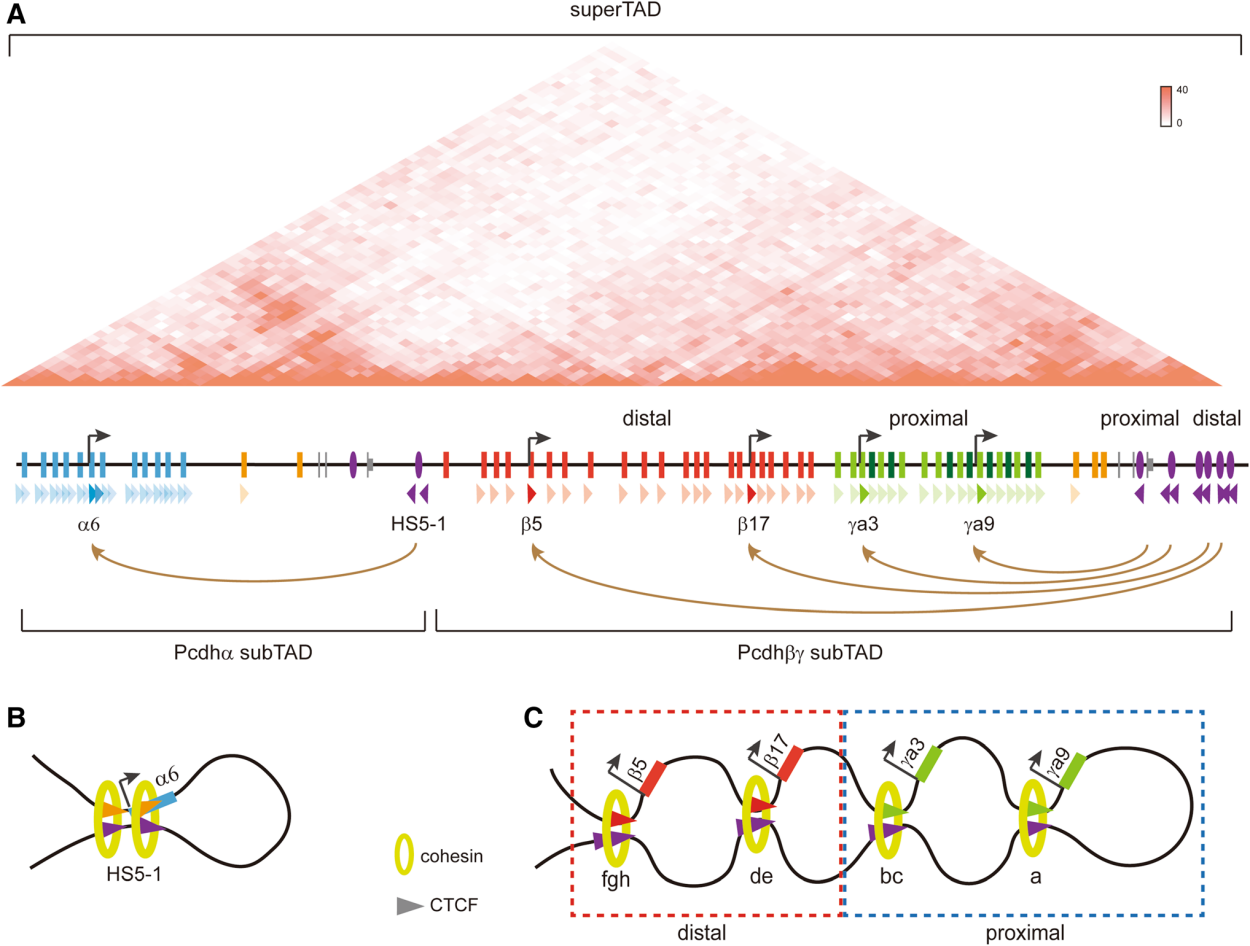
Topological regulation of the clustered *Pcdh* genes in single alleles by tandem CTCF sites. **A** Hi-C map showing the three *Pcdh* clusters are organized into one superTAD composed of two subTADs. In the *Pcdhα* cluster, each promoter-associated CTCF site functions as a topological insulator for all of its upstream genes. In the *Pcdhβγ* clusters, tandem CTCF sites function as topological insulators, resulting in proximal-proximal and distal-distal CBS interactions. **B** HS5-1 forms spatial chromatin contacts with one and only one chosen alternate promoter through CTCF/cohesin-mediated “double-clamp” looping in the *Pcdhα* cluster. **C** Tandem CTCF sites function as topological insulators to balance spatial chromatin contacts and enhancer-promoter selection. The proximal CTCF sites of the super-enhancer form long-distance chromatin interactions with the *Pcdhγ* cluster while the distal CTCF sites of the super-enhancer form long-distance chromatin interactions with the *Pcdhβ* cluster, reminiscent of nested cohesin-extruded loops in the extended “Hulu model” [48].

The three *Pcdh* gene clusters are organized as a large superTAD (super topologically associating domain) which can be divided into two subTADs of *α* and *βγ* (Fig. 2A) [45, 64]. The *Pcdhα* subTAD is formed by long-distance chromatin interactions between tandem arrays of forward CBS elements or CTCF sites of the variable region and the two reverse CBS elements flanking HS5-1 (Fig. 2A). The *Pcdhβγ* subTAD is formed by long-distance chromatin interactions between tandem arrays of forward and reverse CBS elements within the promoter and super-enhancer regions (Fig. 2A). We outline the important role of higher-order chromatin structures in the regulation of clustered *Pcdhs* in this section.

### CTCF Protein as a Key 3-D Chromatin Architect

CTCF (CCCTC-binding factor) is the best-characterized insulator-binding protein in mammals that organizes the 3-D architecture of the genome. It regulates gene expression of the clustered *Pcdh*s through mediating long-range chromatin contacts between remote enhancers and target promoters. The topological chromatin loops between enhancers and promoters are formed by cohesin-mediated active loop extrusion [48]. Cohesin, a ring-shaped complex embracing double-stranded DNA, continuously extrudes chromatin fibers until blocked by CTCF-bound CBS elements. The cohesin loop extrusion brings the two remote DNA fragments with forward-reverse convergent CBS elements into close contact in the 3-D nuclear space [45, 65–67].

There is substantial evidence for a central role of CTCF/cohesin in clustered *Pcdh* gene regulation. First, knockdown of cohesin results in the loss of chromatin loops and downregulation of the clustered *Pcdh* genes [44, 68]. Second, knockdown of CTCF in cell lines also results in the loss of chromatin loops and a significant decrease of *Pcdh* expression levels [44, 68, 69]. Finally, conditional knockout of CTCF in neurons markedly downregulates a staggering 53 out of the total 58 clustered *Pcdh* genes in mice [70], providing strong evidence that CTCF is a master regulator for clustered *Pcdhs* [69].

### Oriented CTCF Sites as Codes of Articulation Joints for Building 3-D Genome Architecture

Initial computational analyses identified a conserved sequence element (CSE), with a highly-conserved CGCT box, located at about the same distance upstream of the translational start codon of each member of the three *Pcdh* clusters (except for *αc2*, *β1*, *γc4*, and *γc5*) [40]. These CSEs were later shown to bind CTCF proteins, and thus are CBS elements (Fig. 1A) [44, 68, 69].

In the *Pcdhα* cluster, there is an additional CBS element located at ~700 bp downstream of the CSE within the coding region of each alternate variable exon (known as eCBS for exonic CBS) (Fig. 1A) [44, 68]. Thus, there are two CBS elements (CSE and eCBS) flanking each *Pcdhα* alternate promoter. However, there is only one CBS element (CSE) associated with the *αc1* promoter and no CBS element associated with the *αc2* promoter (Fig. 1A). Interestingly, the *HS5-1* enhancer is also flanked by two CBS elements, *HS5-1a* and *HS5-1b*, with an intervening distance similar to that between each promoter-flanking CBS pair of CSE and eCBS (Fig. 1A) [44, 68].

All of the CBS elements (CSE and eCBS) flanking the *Pcdhα* promoters are in the forward orientation. By contrast, the two CBS elements (*HS5-1a* and *HS5-1b*) flanking the *HS5-1* enhancer are in the reverse orientation (Fig. 1A). Namely, the CBS elements in the *Pcdhα* promoter and enhancer regions are in the opposite orientation [44]. Forward-oriented CBS elements flanking a *Pcdhα* promoter and reverse-oriented CBS elements flanking the *HS5-1* enhancer interact spatially to form a “double-clamp” transcription hub through CTCF/cohesin-mediated chromatin looping (Fig. 2B) [44, 71].

### CTCF Site Orientation Determines the Directionality of Chromatin Looping

Inversion of the two enhancer CBS elements (*HS5-1a* and *HS5-1b*) in cells and mice by using the CRISPR/Cas9-mediated DNA fragment editing method provides strong evidence for the causality between CBS orientation and chromatin-looping directionality [45, 48]. Specifically, the reverse-oriented CBS elements flanking the *HS5-1* enhancer normally form long-distance chromatin interactions with the forward-oriented CBS elements associated with the upstream *Pcdhα* promoters (Fig. 2A, B) [44]. After inversion by CRISPR DNA-fragment editing [72, 73], however, they no longer form long-distance chromatin interactions with the upstream *Pcdhα* promoters. Strikingly, the inverted CBS elements form long-distance chromatin interactions with the downstream CBS elements [45]. Thus, the relative orientation determines the directionality of long-distance chromatin looping [45]. In addition, spatial chromatin contacts are preferentially formed between forward-reverse CBS elements through CTCF/cohesin-mediated loop extrusion throughout the entire genome [45, 47, 48, 65, 67, 74]. Finally, these experiments also provide strong *in vivo* evidence that enhancers do not function in an orientation-independent manner, at least those associated with CBS [45].

### Tandem CTCF Sites as Genome Topological Insulators

In the *Pcdhβγ* clusters, only a single CTCF site is associated with each variable promoter (except *β1*, *γc4*, and *γc5*) (Fig. 1A) [44, 68, 70]. Similar to the *Pcdhα* cluster, all of the promoter CTCF sites are in the forward orientation in the *Pcdhβγ* clusters. By contrast, the downstream super-enhancer contains several reverse-oriented CTCF sites organized in tandem (Fig. 1A) [45, 46, 48].

Genetic deletion of *HS18-20* (part of the super-enhancer [46]) in mice results in a significant decrease of expression levels of the *Pcdhβ* genes [43]. In addition, deletion or inversion of *HS5-1bL* together with *HS18-20* in mice totally abolishes the expression of all *Pcdhβ* genes, suggesting that these regulatory elements, bypassing the *Pcdhγ* cluster, are enhancers for members of the *Pcdhβ* cluster [43, 47]. However, the expression levels of the *Pcdhγ* genes are mostly unaffected in these deletions, leaving the regulation of the *Pcdhγ* genes an unresolved question [43, 47].

The *Pcdhβγ* genes are topologically regulated by the tandem CTCF sites of the downstream super-enhancer. Specifically, chromosome conformation capture experiments have revealed that the *Pcdhγ* genes are in close spatial contact with the proximal CTCF sites of the super-enhancer (Fig. 2A, C). By contrast, the *Pcdhβ* genes are in close spatial contact with the distal CTCF sites of the super-enhancer (Fig. 2A, C) [48]. This topological regulation solves the long-standing mystery of *Pcdhγ* gene regulation.

These proximal-to-proximal and distal-to-distal topological chromatin regulations were further confirmed by a series of genetic manipulations of the CTCF sites in the super-enhancer *in vivo.* Specifically, when CTCF sites in the super-enhancer are deleted or inverted, the downstream reverse-oriented CTCF sites show increased chromatin interactions with members of the *Pcdhγ* cluster and decreased chromatin interactions with members of the *Pcdhβ* cluster [47, 48]. Thus, tandem CTCF sites function as topological insulators to mitigate the chromatin contacts with and usage of the proximal *Pcdhγ* promoters. In addition, these topological insulators, counter-intuitively, promote chromatin contacts with and the usage of the distal *Pcdhβ* promoters. In conclusion, tandem arrays of oriented CBS elements determine the allocation of spatial resources of enhancers for promoters of both distal and proximal *Pcdh* genes.

### Epigenetic Regulation of Chromatin Loops

Methylation of the CpG dinucleotide within the CGCT box of the CTCF sites of *Pcdh* promoters precludes CTCF binding, suggesting epigenetic regulations of the clustered *Pcdh* genes [44]. In each cell, these CTCF sites are differentially methylated, with one and only one alternate exon being activated through long-range chromatin contacts with the *HS5-1* enhancer (Fig. 2B) [48, 75–77]. In the neuroblastoma cell line SK-N-SH, *Pcdhα* expression levels are inversely correlated with promoter methylation. Specifically, the CBS elements of expressed isoforms are unmethylated and bound by CTCF, but the CBS elements of silenced isoforms are methylated and devoid of CTCF proteins [44]. Consistently, demethylation of CBS elements activates *Pcdhα* gene expression [78]. Finally, recent structural analyses suggest that the addition of a methyl group at the 5^th^ position of cytosine within the CpG interferes with the binding of CTCF zinc finger 3 to the CGCT box [79].

In neurons, the DNA methylation states of the *Pcdh* promoters are also inversely correlated with the transcription states of the *Pcdh* genes. For example, alternate *Pcdhα* genes, which are stochastically expressed by individual Purkinje cells, show mosaic and differential methylation patterns. In contrast, the C-type isoforms, which are constitutively expressed, are hypomethylated [75]. Thus, stochastic expression of *Pcdh* isoforms is probably determined by the DNA methylation in individual neurons.

Recent studies revealed that the eCBS element of each alternate exon is associated with an antisense promoter which transcribes a long non-coding RNA (lncRNA) [78]. Stochastic transcription of this lncRNA extends through the sense promoter, leading to DNA demethylation of the corresponding CBS element. This CBS demethylation then facilitates CTCF binding and subsequent activation of the sense promoter [78]. Interestingly, the promoter activation mediated by antisense lncRNA transcription is only found in alternate but not C-type *Pcdhα* genes. This is consistent with the fact that the C-type *Pcdhα* variable exons do not contain an eCBS element.

### Other Potential Regulatory Proteins

In addition to the architectural proteins CTCF and cohesin, other potential 3-D genome architectural proteins have been shown to regulate expression of the clustered *Pcdh* genes. For example, a protein known as structural maintenance of chromosome hinge domain containing 1 (SMCHD1), which is critically involved in the pathogenesis of facioscapulohumeral muscular dystrophy, antagonizes CTCF in *Pcdh* gene regulation [80]. The SMCHD1 occupancy at *Pcdhα* promoters and enhancers coincides with CTCF sites. Loss of *Smchd1* results in increased CTCF binding to the *Pcdhα* alternate promoters and upregulation of *Pcdh α* and *β* gene expression [80]. However, the underlying mechanism by which SMCHD1 antagonizes CTCF DNA binding remains unknown.

SET domain bifurcated 1 (*Setdb1*) is required for the maintenance of the superTAD structure in *Pcdh* clusters [64]. Conditional knockout of *Setdb1* in forebrain neurons results in the loss of H3K9me3, leading to demethylation of DNA and subsequent recruitment of CTCF to *Pcdh* promoters [64]. The increased CTCF binding strengthens the chromatin interactions between *Pcdh* promoters and enhancers, but weakens the chromatin interactions between the boundaries of the superTAD. Neurons without *Setdb1* lose the stochastic constraint and express increased numbers of *Pcdh* isoforms [64].

Neuron-restrictive silencer factor (*NRSF*) regulates the neuron-restrictive expression of *Pcdhα* through binding to *HS5-1* and *Pcdhα* variable exons [50, 81]. In addition, Wiz (widely-interspaced zinc finger-containing protein) defines cell identity by functioning as a DNA loop anchor in collaboration with CTCF and cohesin [82]. Wiz has been shown to regulate *Pcdhβ* gene expression in mice [83]. Consistently, Wiz proteins are enriched at all of the *Pcdhβ* promoters (except *Pcdhβ1,* which is the only *Pcdhβ* gene with no CTCF site) and at the *HS5-1bL* site of the *Pcdhβγ* super-enhancer [83]. All in all, various transcription factors may regulate the stochastic expression of clustered *Pcdh*s by altering higher-order architectural chromatin loops between enhancers and promoters.

## Mechanisms for Generating Clustered Pcdh Codes of Neuronal Identity

### Combinatorial Expression of Pcdhs as Cell-Surface Identity Codes

Each cortical neuron stochastically expresses up to 2 alternate *Pcdhα* genes, 4 *Pcdhβ* genes, and 4 alternate *Pcdhγ* genes as well as all of the 5 C-type *Pcdh* genes (up to 15 in total) [48, 84]. These combinatorial expression patterns could generate the large number of address codes required for neuronal identity. For example, the 22 encoded Pcdhγ proteins have been predicted to form up to 234,256 distinct tetramers of cell-surface assemblies [85]. In conjunction with the encoded 15 Pcdhα and 22 Pcdhβ proteins, Pcdh proteins could generate the enormous diversity of cell-surface assemblies required for coding single neurons in the brain. We summarize the mechanisms of *Pcdh* promoter choice and expression regulation in this section.

### Establishment and Maintenance of Clustered *Pcdh* Expression Patterns

A remarkable property of the clustered *Pcdh* genes is that their promoter choice is inherited and stably maintained by daughter cells as seen in the SK-N-SH cell line and differentiated neurons [44, 86]. This suggests that, once chosen, the expression patterns of clustered *Pcdh* genes are epigenetically inheritable. In addition, *Pcdh* promoter choice occurs early during the naive-to-primed conversion of ESCs (embryonic stem cells) [86]. The *Pcdh* promoters are modified with both active (H3K4me3) and repressive (H3K27me3) chromatin marks, so called bivalent promoters, in the primed ESCs before being activated. The chosen *Pcdh* genes are then stably inherited by differentiated neurons [86].

**Fig. 3:**
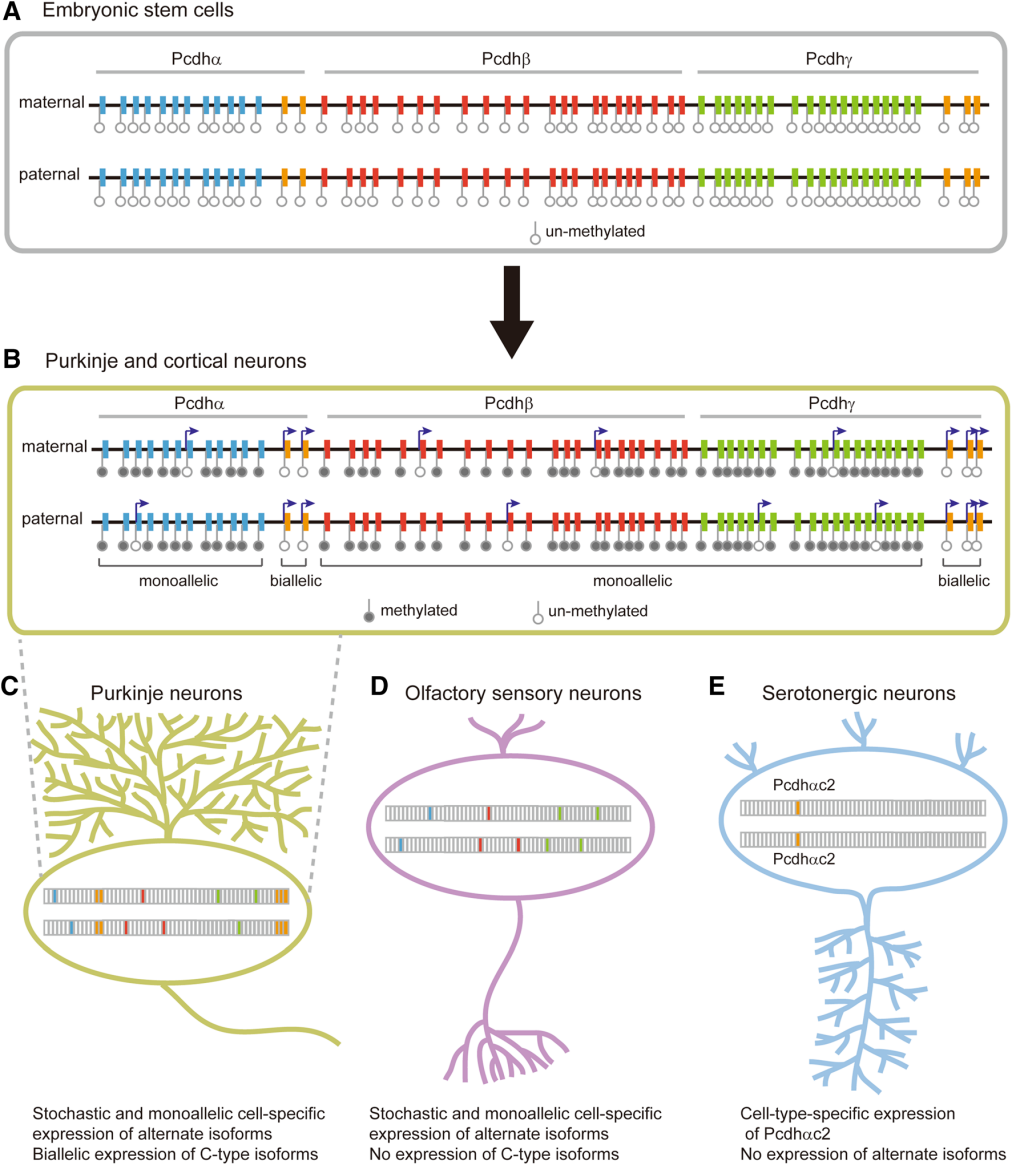
Cell-specific and cell-type-specific expression of clustered *Pcdh* genes. **A** In embryonic stem cells, the ground state of clustered *Pcdh* gene promoters is unmethylated. **B** Gene regulation of the three *Pcdh* clusters by DNA methylation in mature neurons such as Purkinje and cortical cells. **C** In the Purkinje neurons of the cerebellum, alternate genes are stochastically and monoallelically expressed in a cell-specific manner, while C-type genes are constitutively and biallelically expressed presumably in every cell. **D** In olfactory sensory neurons, alternate genes are stochastically and monoallelically expressed in a cell-specific manner, while the C-type genes are not expressed. **E** In serotonergic neurons in the raphe nuclei of the midbrain, only *Pcdhαc2* is expressed in a cell-type-specific manner.

As the methylation states of promoters are inversely correlated with the expression levels of clustered *Pcdh* genes, a fundamental question is how single neurons achieve the stochastic activation of *Pcdh* promoters. On the one hand, stochastic activation of a *Pcdh* promoter could be achieved through demethylation of the chosen target promoter by antisense transcription of lncRNA [78]. On the other hand, this could be achieved through methylation of all of the non-chosen promoters [75]. Consistently, all of the *Pcdhα* alternate promoters are enriched with CTCF in naive ESCs, while only chosen promoters are enriched with CTCF in primed ESCs [86], suggesting hypomethylation-to-hypermethylation conversion of the non-chosen promoters during cellular differentiation. This indicates that the ground state of *Pcdh* promoters is unmethylated or hypomethylated and that the activation of specific promoters requires methylation of all of the other promoters (Fig. 3A, B).

### Cell-Specific and Stochastic Expression of Clustered *Pcdh* Genes

Clustered *Pcdhs* are widely expressed in the developing and adult central nervous systems [11, 34, 35, 42, 53, 87–90]. The expression of members of the *Pcdhα* cluster is highly specific to the central nervous system. While members of the *Pcdh β* and *γ* clusters are prominently expressed in the central nervous system, they are also expressed in several other tissues such as the kidney and lung [87, 89, 91]. Detailed expression patterns of each isoform were initially analyzed by *in situ* hybridization using isoform-specific probes, which showed that they are stochastically expressed in neuronal subpopulations in various brain nuclei or regions [35, 42, 53, 89, 90, 92].

Single Purkinje neurons express alternate members of clustered *Pcdh* genes in a stochastic and monoallelic manner (Fig. 3C) [92–94]. In addition, single cortical neurons also express alternate members of the three *Pcdh* clusters in a similar manner [48, 75, 84]. In the *Pcdhα* cluster, each tandem pair of the promoter CTCF sites (CSE and eCBS) functions as an insulator for all of its upstream *Pcdhα* genes. A single chromatin loop between the *HS5-1* enhancer and a variable promoter determines the expression of the chosen *Pcdhα* gene in each allele (Fig. 2A, B) [48].

In the *Pcdhβγ* clusters, the super-enhancer is composed of four CBS-containing elements. Up to two *Pcdhβ* genes (activated by enhancers with CTCF sites “*de*” and “*fgh*”) and two alternate *Pcdhγ* genes (activated by enhancers with CTCF sites “*a*” and “*bc*”) could be expressed from each allele through nested chromatin loops (Fig. 2A, C) [48].

In olfactory sensory neurons (OSNs), clustered *Pcdh* genes are stochastically expressed, except for the C-types (Fig. 3D) [76]. In addition, diploid chromatin conformation capture of single OSNs has shown that there are significant cell-to-cell heterogeneities of *Pcdh* chromatin architectures and that *Pcdh* enhancers communicate with distinct *Pcdh* promoters in different cells [95]. This may reflect the stochastic *Pcdh* promoter choice. Specifically, each OSN expresses a distinct set of up to 10 alternate *Pcdh* genes, among which 5 are stochastically and monoallelically expressed from each allele (Fig. 2). In summary, these findings suggest that the clustered *Pcdh* genes are stochastically expressed in single neurons of the cerebellum, cerebrum, and olfactory epithelium in a cell-specific manner.

### Cell Type-Specific Expression of Clustered *Pcdh* Genes

All of the C-type *Pcdh* genes appear to be constitutively and biallelically expressed in single neurons of the cerebellum and cerebrum in the mouse brain (Fig. 3B, C) [48, 75, 84, 92–94]. By contrast, none of the C-type *Pcdh* genes is expressed in mouse OSNs (Fig. 3D) [76]. Finally, only *Pcdhαc2* is predominantly expressed in serotonergic neurons (Fig. 3E) [96, 97]. Collectively, these studies suggest that C-type *Pcdh* genes are expressed in a cell-type-specific manner, in stark contrast to the stochastic expression of alternate *Pcdh* genes in the brain.

## Molecular Logic of Neuronal Self-avoidance and Coexistence

### Promiscuous *Cis*-interactions for Diverse Cell-Surface Assemblies

The Pcdhα proteins co-immunoprecipitate with Pcdhγ in cell lysates. In addition, cell-surface delivery of Pcdhα proteins (except for Pcdhαc2) requires the co-expression of Pcdhγ because Pcdhα alone cannot be sufficiently expressed at the plasma membrane [12, 98], suggesting that Pcdhα and Pcdhγ may form heterodimers. Moreover, distinct members interact with each other in membrane fractions [85, 99]. Finally, each member of Pcdhβ or Pcdhγ (except for Pcdhγc4) can form homodimers or heterodimers; however, members of Pcdhα and Pcdhγc4 cannot form homodimers. They can only form heterodimers with Pcdhβ or other Pcdhγ isoforms [12, 100].

**Fig. 4:**
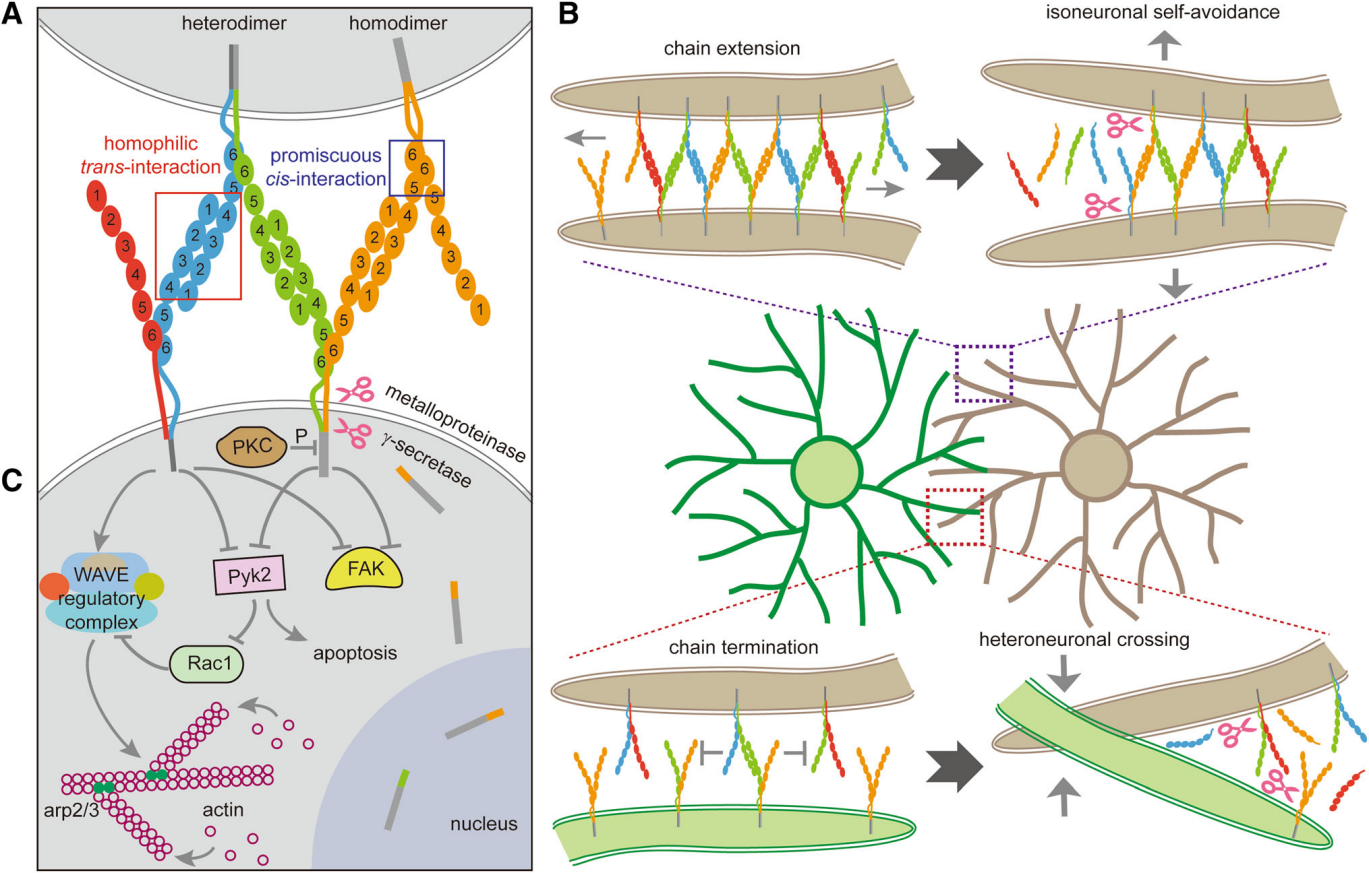
The molecular basis of self-recognition for self-avoidance and non-self coexistence mediated by clustered Pcdhs. **A** Clustered Pcdh isoforms form heterodimers and homodimers in the cell membrane through promiscuous *cis*-interactions between the EC5–EC6 domains of one isoform and the EC6 domain of the other isoform to endow each cell with an identity code. Clustered Pcdh isoforms from different neurites recognize each other through strict homophilic *trans-*interactions of the EC1–EC4 domains in an anti-parallel fashion. **B** Molecular arrangements of an extended self-recognition complex between identical combinatorial profiles expressed on the same neuron, resulting in adhesion-mediated repulsion between sister neurites from single neurons (isoneuronal self-avoidance). Specifically, when the expressed isoforms are the same between two neurites (from the same cell, for example), Pcdh isoforms linearly assemble into parallel arrays through *cis*- and *trans*-interactions to form larger zipper-like lattices between membranes. These structures trigger intracellular signaling and cytoskeletal rearrangement. Subsequent Pcdh cleavage may result in neurite self-avoidance. By contrast, when the two neurites from different neurons stochastically express distinct combinations of Pcdh isoforms, their assembly is interrupted by the mismatched isoforms, as proposed by the isoform-mismatch chain-termination model. This results in heteroneuronal crossing and coexistence. **C** Intracellular signaling of the clustered Pcdhs. Pcdh isoforms are cleaved by metalloproteinase and γ-secretase into an extracellular fragment and an intracellular fragment. The latter may translocate into the nucleus to regulate gene transcription. PKC phosphorylates the intracellular domain of Pcdhγ. Isoforms of *Pcdh α* and *γ* clusters bind and inhibit the activities of Pyk2 and FAK through the Pcdh intracellular domain. The intracellular domain of Pcdhα isoforms recruits the WAVE complex through the WIRS motif and activates actin-filament branching. Pyk2 also inhibits Rac1 and disinhibits the WAVE complex. These intracellular signaling pathways eventually lead to cytoskeletal remodeling and sister-neurite repulsion.

Structural studies support the formation of *cis*-homodimers or *cis*-heterodimers between isoforms of clustered Pcdhs. The *cis*-dimerization requires both EC5 and EC6 domains [13, 60, 101]. Specifically, the Pcdh *cis-*dimer interfaces are asymmetric, with one molecule providing the EC5 and EC6 side of the interface, and the other providing only the EC6 side (Fig. 4A) [13, 60]. Isoforms of Pcdhβ and Pcdhγ (except for Pcdhγc4) form *cis*-homodimers or *cis*-heterodimers in that each isoform can participate as either the EC5–EC6 or EC6 side of the interface [13, 60]. However, isoforms of Pcdhα and Pcdhγc4 can only form *cis*-heterodimers and cannot form *cis*-homodimers because they cannot participate as the EC6 side of the interface. Namely, they participate only as the EC5–EC6 side of the heterodimer interface. They need isoforms of either Pcdhβ or Pcdhγ (also known as carrier isoforms, except for Pcdhγc4) to provide the EC6 side of the heterodimer interface [60].

In summary, clustered Pcdh isoforms appear as a cell-surface repertoire composed of homodimers and promiscuous heterodimers of members of all three Pcdh clusters on the plasma membrane of single neurons [12, 13, 60, 85, 100].

### Homophilic *Trans*-interactions for Self-recognition

Great progress has been made in deciphering the *trans*-interactions of clustered Pcdh proteins for generating cell-recognition specificity. The *trans*-interactions of the Pcdh isoforms have been tested using an efficient cell-aggregation assay by transfecting two cell populations [12, 85, 101]. Different cell populations expressing the same combinations of Pcdh isoforms display strict homophilic interactions and can form cell aggregates, but those expressing different combinations of Pcdh isoforms cannot [12].

All of the clustered Pcdh β and γ isoforms, except for Pcdhγc4, can engage in robust and highly specific *trans*-homophilic interactions in cell aggregation assays. These isoforms are delivered to cell membrane, probably because they can form *cis*-homodimers [60]. Pcdhα (except for Pcdhαc2) and Pcdhγc4, on the other hand, cannot form *cis*-homodimers and cannot be delivered to cell membrane by themselves. Therefore, they cannot induce cell aggregates [12]. Pcdhαc2, however, is unique in that it can induce cell aggregates by itself because it can form *cis*-homodimers and be delivered to cell membrane [12].

The Pcdhα proteins can form *cis-*heterodimers with isoforms of Pcdh β and γ (except for Pcdhγc4). They can be delivered to cell membrane when they are co-expressed with Pcdh β or γ isoforms. Therefore, Pcdhα (except for Pcdhαc1) does induce cell aggregates through homophilic *trans-*interactions when co-expressed with Pcdh β and γ isoforms (except for Pcdhγc4). Finally, homophilic interactions are abolished when there is a single mismatched isoform between the two transfected cell populations [12].

Structural analyses revealed that the *trans*-homophilic interactions are mediated by EC1–EC4 in an antiparallel manner. These *trans-*interactions form a zipper-like ribbon structure in apposed plasma membranes. Specifically, the EC1, EC2, EC3, and EC4 of one isoform at a cell surface interact with the EC4, EC3, EC2, and EC1 of the same isoform from the apposed cell surface, respectively [13, 101–105]. Among the six EC domains of clustered Pcdhs, EC2 and EC3 have been positively selected for diversity during evolution and are thus the most diversified ECs in amino-acid residues [52]. Consistently, they determine the stringent specificity of *trans*-homophilic interactions [12, 85, 104].

### The Chain-Termination Model for Non-self Discrimination

The crystal structure of the full-length extracellular domain of Pcdhγb4 reveals a zipper-like lattice through *cis-*interactions mediated by EC5–EC6/EC6 and *trans*-interactions mediated by EC1–EC4 [13]. When tethered to liposomes, Pcdh extracellular domains spontaneously assemble into zipper-like linear arrays through *trans*-homophilic interactions between Pcdh dimers [13]. These linear assemblies extend through the contacted membranes as a chain to form a larger lattice (Fig. 4B). In this chain termination model, once a certain size threshold is reached, the assemblies trigger intracellular Pcdh signaling pathways to regulate various cellular behaviors such as repulsion. By contrast, when mismatched isoforms are incorporated, the Pcdh chain extension terminates and the lattice size cannot reach the presumed signaling threshold (Fig. 4B) [13, 101]. This isoform-mismatch chain-termination model can explain the recognition initiation process of self and non-self discrimination mediated by the extracellular domains of clustered Pcdhs.

### Intracellular Signaling of Clustered Pcdhs Leads to Cytoskeletal Rearrangement and Morphological Remodeling

The intracellular domains of the Pcdhα and Pcdhγ isoforms contain a respective common membrane-distal region encoded by constant exons that is shared by all isoforms from the same cluster [11, 14]. The Pcdhα and Pcdhγ isoforms are cleaved by metalloproteinase and subsequently by γ-secretase to generate a soluble extracellular fragment and an intracellular fragment that may function locally or translocate into the cell nucleus [106–109]. This proteolytic process requires endocytosis and is regulated during animal development and neuronal differentiation [110].

The Pcdhα and Pcdhγ proteins can bind and inhibit two cell-adhesion kinases, FAK (focal adhesion kinase) and Pyk2 (proline-rich tyrosine kinase 2), through the cytoplasmic domain (Fig. 4C) [111]. In the mouse hippocampus and cortex, Pcdhα and Pcdhγ regulate dendritic arborization and spine morphogenesis through inhibiting Pyk2 and FAK activity [112–114]. Knockout or knockdown of *Pcdhα* in hippocampal neurons results in the phosphorylation and activation of Pyk2 [113]. The activation of Pyk2 inhibits Rac1, leading to defects in dendritic and spine morphogenesis. Consistently, knockdown of *Pyk2* or overexpression of *Rac1* rescues the phenotype caused by *Pcdh α* or *γ* knockdown [113]. *Pcdhγ* knockout induces extensive neuronal apoptosis in the spinal cord [6], which could be related to aberrantly up-regulated Pyk2 activity. Consistent with this, over expression of Pyk2 also induces apoptosis [111]. Together, these data suggest that diverse extracellular signals acting on different Pcdhα and Pcdhγ isoforms converge into the same intracellular pathways through common downstream effectors of Pyk2 and FAK (Fig. 4C).

The common intracellular domain of Pcdhα isoforms, but not Pcdhγ isoforms, contains a conserved peptide WIRS (WAVE-interacting receptor sequence) motif that interacts with the WAVE (Wiskott-Aldrich syndrome family verprolin homologous protein) regulatory complex (WRC) to modulate cytoplasmic actin assembly (Fig. 1B) [115, 116]. Specifically, Pcdhα isoforms (except for Pcdhαc2) regulate cytoskeletal dynamics during cortical neuron migration and dendrite morphogenesis through the WAVE regulatory complex (Fig. 4C) [116]. Overexpression of Pcdhα isoforms (except for Pcdhαc2) rescues the migration defects caused by *Pcdhα* knockdown and the rescue is abolished by WIRS mutation. In addition, overexpression of WRC subunits also rescues the migration defects of *Pcdhα* knockdown [116]. Given that Pcdhα forms *cis*-heterodimers with Pcdh β or γ on the cell surface (Fig. 4A), the Pcdh β and γ isoforms may also modulate the WAVE complex through interacting with Pcdhα (Fig. 4C). Specifically, Pcdh β and γ proteins, together with Pcdhα, may regulate neuronal morphogenesis and dendrite self-avoidance through WAVE dynamics and cytoskeletal rearrangements (Fig. 4C). In summary, the establishment and maintenance of neuronal connectivity and self-avoidance likely require coordinated collaborations between members of all three *Pcdh* gene clusters.

## Concluding Remarks and Future Perspectives

In the central nervous system, individual neurons stochastically express combinatorial sets of clustered Pcdhs. These Pcdh expression profiles constitute diverse cell-surface identity codes through *cis*-promiscuous pairing and discriminate self from non-self through strict *trans*-homophilic interactions. Their tremendous diversity is generated by intriguing 3-D genome architecture, stochastic promoter choice balanced by topological insulators, long-range spatial chromatin contacts between distal enhancers and target promoters, and alternative splicing.

Elucidating the regulatory mechanisms of clustered *Pcdhs* in different cell types throughout the nervous system will be of great importance in deciphering the molecular basis underlying neural-circuit coding. Several lines of investigation of the *Pcdh* clusters have provided deep insights into various aspects of gene expression mechanisms, from 1-D genomic organization to 2-D epigenetic regulation and 3-D chromatin architecture. However, many important questions remain unanswered. For example, when are Pcdh isoforms chosen to be expressed in neuronal progenitor cells during brain development? What is the mechanistic basis for the epigenetic memory of clustered *Pcdh* expression profiles? What are the mechanistic differences between the regulation of expression of alternate and C-type isoforms? How do serotonergic neurons selectively express only the *Pcdhαc2* gene in a cell-type-specific manner? Finally, how do clustered Pcdhs collaborate with other families of cell-adhesion proteins to specify synaptic connectivity? Answering these questions about neural coding mechanisms in the brain requires interdisciplinary endeavors in the future.
